# Enactive Memory

**DOI:** 10.3389/fpsyg.2020.00114

**Published:** 2020-02-04

**Authors:** Denis Brouillet

**Affiliations:** EPSYLON Laboratory (EA 4556), University Paul Valéry, Montpellier, France

**Keywords:** cognition, memory, enaction, limb movements, oculomotor movements

Since its birth in 1953, cognitive psychology has been dominated by a conception of cognition as computations of mental representations of the world (see Fodor, 1983). But in the last three decades a radical change has occurred: cognition is now viewed as emerging from cognitive processes closely intertwined with actions performed in a specific environment (Varela et al., 1991).

The first consequence of this view is that the ultimate aim of cognitive processes is not to recover representations or to construct new representations of a pre-existent world, but rather to create the world in which people act. According to this view the perceived world is a construction linked to the mutual dependence between a cognitive agent and the world in which s/he evolves; that is to say, the world is enacted by one's actions. The second consequence is that for the enactive approach, perception, action, and cognition are in substance intrinsically linked. Indeed, it emphasizes the interdependence of two points: (a) perception consists of perceptually guided action; and (b) cognitive structures emerge from recurrent sensorimotor patterns that guide the action perceptually (Varela et al., 1991, p. 173).

The idea that perception, action, and cognition are intrinsically linked was already supported by the ecological theory of perception (Gibson, 1979), for which action is performed through the exploratory activity of vision. The sensorimotor approach to perception (for a review, O'Regan, 2011) establishes the same link between perception, action, and cognition. The action is essential for perceiving invariants, updating it through what are called sensorimotor contingencies.

If the idea that our perceptions are enacted is relatively easy to conceive, it is otherwise with memory. Indeed, considering that our memories may be enacted is to say that they are the product of the actions we take to account for them in a given situation. In other words, this means accepting that our memories are no longer considered as the recovery, *sensu stricto*, of an event that happened in the past, but rather as the product of a cognitive elaboration constructed here and now. To paraphrase Neisser (1996), memories are a kind of reflection of the dynamics of cognition; they emerge from this dynamic. So, memories are not static; based on the synergy between previous experiences and present experience, they are continuously revised. Adopting this point of view would be tantamount to breaking with the conception of memory that is widely shared and defended by classical models of memory (from the Atkinson and Shiffrin's model, 1968, up until the *SPI* model, Tulving, 1995).

Nevertheless, alternative models of memory, proposed since 1980s, appear to be compatible with an enactive approach to memory. These models, called *single memory models*, postulate that the experiences of our interactions with the environment are stored in a multi-sensory way and distributed across the entire brain (Hintzman, 1986; Whittlesea, 1987; Versace et al., 2014). Here, memory traces reflect the processing episode and retrieval is the result of a coupling between the recovery situation and a set of episodic traces activated on the basis of their similarity to the characteristics of the recovery episode (see, the *synergistic ecphory process* modeled in MINERVA, Hintzman, 1986). The consequence, the memories are not recovered; they emerge in the present situation.

By including an *integration process*, considered as a dynamic mechanism, the Act-In model (Versace et al., 2014) has enhanced the MINERVA model. In Act-In, activations occur continuously in parallel with the computation of similarities; and while the integration mechanism is running, traces are modified both in a proactive manner (the content of a trace depends on the content of previous traces that are activated), and in a retroactive manner (the content of a trace modifies the content of previous traces). Therefore, storage and recovery cannot be considered separately. Moreover, since the integration mechanism is dynamic and non-linear, this means that memories are not simply the sum of the components that make up the activated traces; they are a new entity in its own right, which contains more information than the sum of its components.

More recently, the ATHENA model (Briglia et al., 2018), referring to the enactivist idea that the experience of seeing occurs when an organism perceives sensorimotor regularities or sensorimotor contingencies, proposes that what is learned are the covariances between the present sensorimotor information processing and past sensorimotor information processing. Consequently, in ATHENA, memory does not remember recoverable contents but reusable processes (Brouillet and Versace, 2019). So, the ATHENA model shares the radical enactive account of memory that assumes that remembering is not access to or retrieval of contents (Hutto and Peeters, 2018).

In addition to these models suggesting that an enactive approach of memory is a viable option, there are some experimental studies that support the role of action in the recovery of memories^1^.

Using a categorization task, Camus et al. (2018) showed that the action performed in a categorization task affected the results, and that the effect depended on the type of action performed. In addition to demonstrating a classic motor compatibility effect, the results showed that the amplitude of this effect depended on the relationship between stimulus and response acquired during the acquisition phase. This result is particularly interesting for our purpose because it confirms, on the one hand, that there is a memory of the consequences of our actions (see also Brouillet et al., 2015); and on the other, that the action one takes here and now to provide an answer has an effect on memory performance.

Brouillet et al. (2016) investigated whether the action performed at the recovery stage, could affect memory performance. The results showed an effect of motor compatibility. The memory judgment was based on the motor compatibility between on the one hand the actual gesture performed to respond, and on the other, the potential gesture initiated by the visual stimulus. Thus, the resonance of the actual gesture performed to respond with the potential gesture related to the stimulus, or more precisely their directional compatibility, influenced memory performance.

Brouillet et al. (2017) went on to test the idea that it might be the motor resonance itself that was behind their results, independent of any link that might exist between the action to be performed and the item to be recognized. For this, they used the well know effect of laterality (i.e., easiness performing a movement in ipsilateral space than contralateral space). The results showed an effect of motor resonance that was heightened in the right index finger condition (all the participants were right-handed). Thus, when the action to type the two stimuli that appeared before the word to be recognized, required a movement toward the space corresponding to their ipsilateral space, the participants had a recognition score higher than their contralateral space. This effect was observed for both old and new words. The authors concluded that it was subjective perception of the motor resonance (so-called motor fluency) linked to the action and the space of its realization that was the cause of the results observed.

If the action carried out by a limb enact memory, several studies showed that another part of our body that makes movements, without our being aware of it, the eyes, also enact memory.

The study by Kosslyn (1994), showed that eye movements are motor information that can be used to remember images. Christman et al. (2003) showed that performance on a recognition test was dependent on the eye movements made just before the task. Participants were better able to discriminate between old and new words after horizontal eye movements than after vertical eye movements or no eye movement. In a subsequent study, the authors observed that for memory of autobiographical events, horizontal eye movements almost doubled participants' discrimination abilities by increasing the rate of correct recognitions and decreasing the rate of false hits. Brunyé et al. (2009) found similar results using shapes and spatial positions in a recognition task. In addition, the authors showed that this effect was observed only where the movements occurred before the recovery phase and not before the learning phase. Moreover, the effect was amplified when the participants were right-handed. Demichelis et al. (2013) investigated whether, when attempting to restore walking a path learned on a map from our memory, we remember it by simulating the ocular exploration that we originally used to learn it. In one hand the results showed that horizontal movements being more efficient than vertical ones, in other hand, they suggest that the participants had mentally repeated their previous ocular exploration of the map, and that it is this oculomotor memory that was used to perform the locomotor memory task.

In our view, all these findings constitute a strong argument in favor of enacted memory. Indeed, they share with what is the core of enactivism (see, Di Paolo et al., 2010, p. 36–44): the sensorimotor couplings, the role of body and agent, the generation of cognition through action, the emerging character of knowledge, the importance experience (past and present).

But this is not without consequences firstly for our way of conceiving both memory and cognition, secondly for our approach to aging and handicap generally. On the one hand, memory is not a library of memories, even if they are episodic; on the other, memory is not the memory of information that enters the system, but the memory of our actions. However, this does not mean the memory of our actions, strictly speaking, but rather the traces of what has been enacted by our actions. That is to say, the enacted process itself, its phenomenological consequences and its product. Finally, the enactive approach had led questioning the conception of aging considered as only a decline of cognitive functions. Less efficient sensory and motor skills can explain several effects linked to aging, in particular those on memory (Vallet, 2015; Costello and Bloesch, 2017). Moreover, consideration of body specificity can affect clinical evaluation. For example, Turo et al. (2019) showed that spatial working memory (Corsi task) is sensible to manuospatial compatibility (i.e., compatibility between the hand used to the practioner to point the blocs and the dominant hand of the patient).

## Author Contributions

The author confirms being the sole contributor of this work and has approved it for publication.

### Conflict of Interest

The author declares that the research was conducted in the absence of any commercial or financial relationships that could be construed as a potential conflict of interest.

## Footnotes

^1^This is the role of action; that is, how memories are dependent on action and not the memory of actions, which is very well-documented (see Zimmer et al., 2001).

## References

[B1] Atkinson R. C. Shiffrin R. M. (1968). Human memory: A proposed system and its control processes, in The Psychology of Learning and Motivation: Advances in Research and Theory, Vol. 2, eds Spence K. W. Spence J. T. (New York, NY: Academic Press, 89–195.

[B2] Briglia J. Servajean P. Michalland A. H. Brunel L. Brouillet D. (2018). Modeling an enactivist multiple-trace memory. ATHENA: a fractal model of human memory. J. Math. Psychol. 82, 97–110. 10.1016/j.jmp.2017.12.002

[B3] Brouillet D. Brouillet T. Milhau A. Heurley L. Vagnot C. Brunel L. (2016). Word-to-picture recognition is a function of motor components mappings at the stage of retrieval. Int. J. Psychol. 51, 397–402. 10.1002/ijop.12210 27585735

[B4] Brouillet D. Milhau A. Brouillet T. Servajean P. (2017). Effect of an unrelated fluent action on word recognition: a case of motor discrepancy. Psychon. Bull. Rev. 24, 894–900. 10.3758/s13423-016-1160-0 27612859

[B5] Brouillet D. Vagnot C. Milhau A. Brunel L. Versace R. Rousset S. (2015). Sensory-motor properties of past actions bias memory in a recognition task. Psychol. Res. 79, 678–686. 10.1007/s00426-014-0600-6 25081346

[B6] Brouillet D. Versace R. (2019). The nature of the traces and the dynamics of memory. Psychol. Behav. Sci. 8, 151–157. 10.11648/j.pbs.20190806.12

[B7] Brunyé T. T. Mahoney C. R. Augustyn J. S. Taylor H. A. (2009). Horizontal saccadic eye movements enhance the retrieval of landmark shape and location information. Brain Cogn. 70, 279–288. 10.1016/j.bandc.2009.03.003 19346050

[B8] Camus T. Hommel B. Brunel L. Brouillet T. (2018). From anticipation to integration: the role of integrated action-effects in building sensorimotor contingencies. Psychon. Bull. Rev. 25, 1059–1065. 10.3758/s13423-017-1308-6 28537007

[B9] Christman S. D. Garvey K. J. Propper R. E. Phaneuf K. A. (2003). Bilateral eye movements enhance the retrieval of episodic memories. Neuropsychology 17, 221. 10.1037/0894-4105.17.2.221 12803427

[B10] Costello M. C. Bloesch E. K. (2017). Are older adults less embodied? A review of age effects through the lens of embodied cognition. Front. Psychol. 8:267. 10.3389/fpsyg.2017.00267 28289397 PMC5326803

[B11] Demichelis A. Olivier G. Berthoz A. (2013). Motor transfer from map ocular exploration to locomotion during spatial navigation from memory. Exp. Brain Res. 224, 605–611. 10.1007/s00221-012-3336-9 23223779

[B12] Di Paolo E. Rohde M. De Jaegher H. (2010). Horizons for the enactive mind: values, social interaction, and play, in Enaction: Towards a New Paradigm for Cognitive Science, eds Stewart J. Gapenne O. Di Paolo E. A. (London: Bradford Book; Cambridge MIT Press, 32–87.

[B13] Fodor J. (1983). The Modularity of Mind. Boston, MA: MIT Press.

[B14] Gibson J. J. (1979). The Ecological Approach to Visual Perception., Boston, MA: Houghton Mifflin.

[B15] Hintzman D. L. (1986). “Schema abstraction” in a multiple-trace memory model. Psychol. Rev. 93, 411–428. 10.1037/0033-295X.93.4.411

[B16] Hutto D. D. Peeters A. (2018). The roots of remembering: Radically enactive recollecting, in New Directions in the Philosophy of Memory, eds Michaelian K. Debus D. Perrin D. (New York, NY: Routledge, 97–118.

[B17] Kosslyn S. M. (1994). Image and Brain. The Resolution of the Imagery Debate. Cambridge MA: MIT Press.

[B18] Neisser U. (1996). Remembering as doing. Behav. Brain Sci. 19, 203–204. 10.1017/S0140525X00042308

[B19] O'Regan J. K. (2011). Why Red Doesn't Sound Like a Bell: Understanding the Feel of Consciousness. Oxford: Oxford University Press.

[B20] Tulving E. (1995). Organization of memory: Quo vadis?, in The Cognitive Neurosciences, ed Gazzaniga M. S. (Cambridge, MA: The MIT Press, 753–847.

[B21] Turo S. Collin F. Brouillet D. (2019). The importance of the body-specificity in the evaluation of visuospatial working memory. aging, neuropsychology and cognition, in Agir pour connaître, ed Brouillet D. (Grenoble: Presses Universitaires de France), 79. 10.1080/13825585.2020.1799925 32762528

[B22] Vallet G. T. (2015). Embodied cognition of aging. Front. Psychol. 6:463. 10.3389/fpsyg.2015.00463 25932019 PMC4399201

[B23] Varela F. J. Thompson E. Rosch E. (1991). The Embodied Mind: Cognitive Science and Human Experience. Mind. Boston, MA: MIT Press.

[B24] Versace R. Vallet G. T. Riou B. M L. Labeye E. Brunel L. (2014). ACT-IN: an integrated view of memory mechanisms. J. Cogn. Psychol. 26, 280–306. 10.1080/20445911.2014.892113

[B25] Whittlesea B. W. A. (1987). Preservation of specific experiences in the representation of general knowledge. Cognition 13, 3–17. 10.1037/0278-7393.13.1.3

[B26] Zimmer H. D. Cohen R. L. Guynn M. J. Engelkamp J. Kormi-Nouri R. Foley M. A. (2001). Memory for Action: A Distinct Form of Episodic Memory? New York, NY: Oxford University Press.

